# Inertial Motion Capture Costume Design Study

**DOI:** 10.3390/s17030612

**Published:** 2017-03-17

**Authors:** Agnieszka Szczęsna, Przemysław Skurowski, Ewa Lach, Przemysław Pruszowski, Damian Pęszor, Marcin Paszkuta, Janusz Słupik, Kamil Lebek, Mateusz Janiak, Andrzej Polański, Konrad Wojciechowski

**Affiliations:** 1 Institute of Informatics, Silesian University of Technology, Akademicka 16, 44-100 Gliwice, Poland; Agnieszka.Szczesna@polsl.pl (A.S.); Przemyslaw.Skurowski@polsl.pl (P.S.); Przemyslaw.Pruszowski@polsl.pl (P.P.); Damian.Peszor@polsl.pl (D.P.); Marcin.Paszkuta@polsl.pl (M.P.); Andrzej.Polanski@polsl.pl (A.P.); 2 Institute of Mathematics, Silesian University of Technology, Kaszubska 23, 44-100 Gliwice, Poland; Janusz.Slupik@polsl.pl; 3 Polish-Japanese Academy of Information Technology, Koszykowa 86, 02-008 Warsaw, Poland; kamil.lebek@gmail.com (K.L.); mjaniak@pjwstk.edu.pl (M.J.); Konrad.Wojciechowski@polsl.pl (K.W.)

**Keywords:** wearable sensor system, IMU sensor, motion capture, system validation, orientation estimation

## Abstract

The paper describes a scalable, wearable multi-sensor system for motion capture based on inertial measurement units (IMUs). Such a unit is composed of accelerometer, gyroscope and magnetometer. The final quality of an obtained motion arises from all the individual parts of the described system. The proposed system is a sequence of the following stages: sensor data acquisition, sensor orientation estimation, system calibration, pose estimation and data visualisation. The construction of the system’s architecture with the dataflow programming paradigm makes it easy to add, remove and replace the data processing steps. The modular architecture of the system allows an effortless introduction of a new sensor orientation estimation algorithms. The original contribution of the paper is the design study of the individual components used in the motion capture system. The two key steps of the system design are explored in this paper: the evaluation of sensors and algorithms for the orientation estimation. The three chosen algorithms have been implemented and investigated as part of the experiment. Due to the fact that the selection of the sensor has a significant impact on the final result, the sensor evaluation process is also explained and tested. The experimental results confirmed that the choice of sensor and orientation estimation algorithm affect the quality of the final results.

## 1. Introduction

The full-body motion capture technology has applications in various domains, including virtual reality [1], athletic training [2], biomedical engineering [3] and rehabilitation [4,5]. The demand for rehabilitation services and the resulting demand for systems capable of body movement monitoring continue to grow due to the increasing population of ageing people. Remote measurements of human subjects can improve the general health and the quality of life or limit the overall healthcare costs. The user-worn inertial measurement units (IMUs) has been proven to be suitable for unrestrained tracking of body segments’ orientations because they are small, light, affordable and completely self-contained [3,6,7,8,9].

Commonly used IMUs are composed of accelerometers, gyroscopes, and magnetometers, and are characterised by high measurement noise, incorrect scaling and biasing. The bulk of the research concerning IMU sensors has been conducted to reconstruct trajectories or to estimate sensor orientations by the development of efficient filters and algorithms designed to overcome the mentioned sensors’ flaws [10,11,12,13,14,15].

The presentation of IMU-based motion capture systems is mainly focused on their specific applications, rather than its architecture and basis of technical choices. Such systems are usually validated by comparison of reconstructed segment orientations with a marker-based optical system, which is characterised by high accuracy [6,16].

In [5] the authors present a full body sensing system for monitoring the daily-life activities of stroke patients. The position and orientation of each body segment are reconstructed using the commercial Xsens MoCap Engine [9], which provides some degree of performance, however, it also restricts the choice of hardware and methods at the various stages of sensor data collection and processing. The Xsens MVN motion capture suit is presented in [9]. It is an easy-to-use commercial system for full-body human motion capture. However, it can only be evaluated as a complete product. There is no full information about all the operations made to the raw sensor data. In [17], the design of a low-weight and low-power inertial motion capture suit is presented. The authors focus on reducing the energy consumption used by their system. Some parts of the system are designed using the standard solutions which were not subjected to an individual assessment. In [18] the authors present Pedalvatar: a low cost IMU based full body motion capture system; a foot - rooted kinematic model is used. The system can capture full body motion in real-time as long as there is at least one static foot at each time step. As in previous work [17], the authors focus on a particular problem, limiting choice and assessment of the remaining parts of the system. Next works present IMU-based systems that are suitable only for specific applications. For example [3] presents a system for gait training, and [16] describes a system for studying human postural stability onboard. Inertial navigation systems (INS) are based also on IMU sensors with aiding devices (like GPS, barometer sensors). Such systems usually allow to track orientation, position and velocities of one rigid-body (for example vehicle) or multi-body configuration [19,20,21,22].

The fact that we want to point out in this study is that the motion capture system is a set of equally important components. The final quality of motion reconstruction arises from the quality of all subsequent elements. A poor outcome of the orientation estimation algorithm may be caused by e.g., an incorrect setting of the IMU on the segment, rather than by flaws of the algorithm itself. In [23] the authors show that the IMU-to-segment orientation errors have the most severe influence on the estimated orientations and that these errors should be considered during the design or selection of the estimation algorithm. Such dependencies imply the need for the evaluation mechanisms of the different system stages during and after the system design.

In this paper, a new full-body inertial motion capture system is presented. Our contribution is focused on the design study of the individual components of the motion capture system. The two key steps of the system design are explained: sensors evaluation and algorithms for orientation estimation. The results obtained in the experiments have demonstrated the importance of the choice of sensor and orientation estimation algorithm and its influence on the final results. The selection of the sensor also has a significant impact on the sensor evaluation process. For the sensor with better parameters, we obtain a smaller error for the estimated orientation.

The remainder of this paper is organised as follows. We present the details of our system in Section 2. Section 3 presents notation used further in this study. The experimental part is two-fold, in each part relevant theoretic background, experimental results and conclusions are provided. Section 4 explains sensor evaluation process employed by the authors. Section 5 shows the orientation estimation algorithms. Finally, Section 6 briefly concludes the paper.

## 2. System Overview

**Figure 1:**
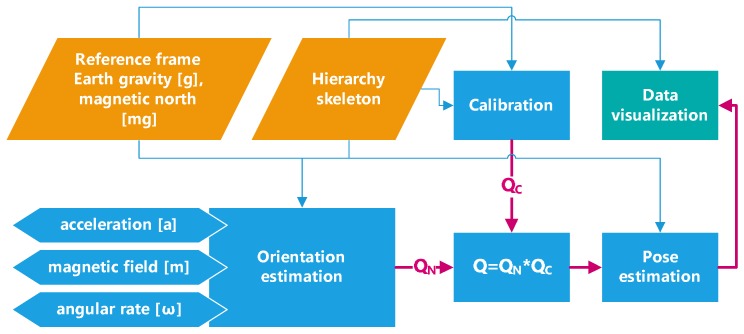
Data processing pipeline.

In this section, a scalable, wearable multi-sensor system for motion capture is introduced. The system is based on body sensor networks (IMU sensors are attached to each segment that should be tracked) integrated with distributed data processing and control software, namely the Multimodal Data Environment (MDE) (developed in Human Motion Lab, PJATK, Poland [24,25]). Currently, MDE enables the processing of simultaneous motion of up to 50 people, who can have a different number of IMUs placed at various body segments. A schema for a data processing pipeline of the system is presented in Figure 1.

**Figure 2:**
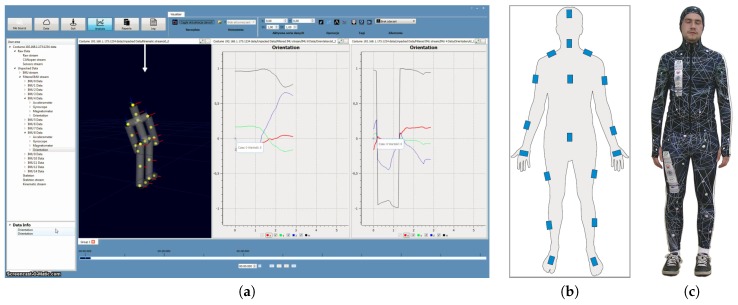
System components: (**a**) MDE software screen-shot; (**b**) sensor locations; (**c**) whole suit.

First, raw sensors data are transmitted to MDE from the Body System (full-body costume with IMU sensors sewn into it). After that, the sensor frame with respect to the global (earth-fixed) reference coordinate system $Q_{N}$ is estimated. Subsequently, the relative rotations $Q_{C}$ between the sensors and the segments and also between the earth frame and the global scene frame are determined during the calibration step. Next, the knowledge of the attachments between the segments is applied to improve the accuracy of the estimated data at the level of the entire skeleton. In the end, the 3D animation Visualizer obtains the processed data (local segments’ orientation), and the entirety of the collected data can be viewed and visualised in a chart form (Figure 2a). At each stage of the pipeline, the data concerning hierarchy skeleton are available to use. During data processing, all orientations are represented by unit quaternions.

The system architecture based on the dataflow programming paradigm makes it easy to add, remove and replace data processing steps. This feature makes our system easy to test and expand, ensuring its relevance in the face of continued research in the associated fields.

### 2.1. Hardware

Two versions of the Body System were developed for our inertial motion capture outfit.

The first version was developed using the wired CAN bus communication that is successfully used in the automotive industry. In this solution, IMUs developed by the authors are used [26]. The devices are based on an ARM processor and a low-cost MEMS sensors including 3-axis accelerometer, 3-axis gyroscope and 3-axis magnetometer. The primary advantage of the CAN bus based solution is the easier synchronisation of the multiple sensors. On the other hand, this solution is not without its drawbacks. It has a bandwidth that is limited by the CANopen protocol specification and requires the use of special multicore elastic wires that provide the communication between sensors and the system hub that is responsible for communicating with the end device. The system hub acts as a server for the UDP endpoints that are allowed to connect to the particular port. The device is responsible for the sensors synchronisation and data acquisition.

In the second version of the developed system (Figure 2c), to ensure the high bandwidth necessary to transmit large amounts of data from multiple sensors, the CAN interface was replaced with the wireless communication. Some of the modern communication modules that are based on the ARM microprocessors offer the free CPU resources that can be used to create the necessary logic. Most of them offer popular communication interfaces such as I2C, SPI, and USART that can be used to communicate with peripherals. The modern, energy-efficient WiFi module was used to create the platform for the wireless IMU data transfer in the second version of the Body System. The sensor communicates with the microprocessor using serial communication. The module is configured as a client and can connect to the wireless network with a specified SSID. The MAC addresses are used as the sensors’ ID numbers and the DHCP server is running on the access point. The sensors obtain an IP address from the DHCP and continuously send the data frames marked with timestamps to the server at the defined sampling rate.

### 2.2. Software

A software application, Multimodal Data Environment (MDE) has been built to visualise, analyse and control the acquisition and monitoring process of human motion [24,25]. It supports processing multimodal data from any number of measurements synchronously. The process of estimating local orientations of body segments from raw sensor data is performed by MDE plug-in, Motion Data Editor (IMU-MDE).

A hierarchical human model has been developed and implemented in a way that allows flexible modelling of any kinematic structure and free parameterization of the model body shape. The length of each segment can be provided, so that the body shape would be represented properly, it also allows for a quick adjustment for the tracked human by entering the length of the shoe and the height. The other segments’ measurements are not necessary in case of a quick adjustment, as they can be calculated on the basis of an anthropometric model, a linear approximation of ANSUR data [27].

The modular architecture of the system allows an effortless introduction of new orientation estimation algorithms. As part of the design study, three selected algorithms (in quaternion version) have been implemented. The test results are shown in Section 5.2.

The output signal from the IMU is based on the sensor’s coordinate system. The orientation estimation stage of the system pipeline returns the orientation of the sensor in the Earth-fixed coordinate system. Due to the fact that it is necessary to express the signal in the body segment coordinate frame and one cannot assume that the sensors are perfectly aligned with the segments to which they are attached, the sensor must be subjected to the step of calibration. The calibration process consists of two parts:

- estimation of the orientation of the earth-fixed frame with respect to segment’s reference frame (scene frame);
- estimation of the exact orientation of each of the sensors with respect to the segment.

In our system, we propose an undemanding, simple action for a root body segment for the purpose of the first estimation. We assume that the “up” axis of both systems are aligned, so we only need to find a rotation from the north vector to the human forward vector, which is calculated from the predefined motion (leaning forward). A predefined posture is used to map the sensor frame on the segment frame: the T-pose (upright with arms horizontally and thumbs forward) or the N-pose (arms neutral besides body). The rotation of the sensor with relation to the body segment is determined by matching the orientation of the sensor in an *a priori* known pose with the known orientation of each segment in this pose [9]. The combination of these two rotations $Q_{C}$ is used as a correction for the calculation of the global segments’ orientation *Q* from the global sensor’s orientation $Q_{N}$.

A pose estimation is achieved with the use of the information about the skeleton and the orientations of the individual segments. Also, it is possible to apply kinematic corrections calculated on the basis of the hierarchy of the skeleton and the anthropometric model. Additional restrictions based on the body joints construction can also be imposed on the calculated hierarchy.

## 3. Notation and Measurements

The modelling of motion of a moving body involves introducing coordinate systems (frames). In this paper, we will use two coordinate systems. The first one is the Earth-fixed coordinate system, called the navigation frame, with the axis pointing north ($x^{N}$), east ($y^{N}$) and up ($z^{N}$). The second one is the coordinate system related to the moving sensor, denoted by ($x^{S}$, $y^{S}$, $z^{S}$) with the origin at the triaxial gyroscope and axes pointing along the gyroscope axes. We will also use abbreviations *N* coordinate frame and *S* coordinate frame for navigation and sensor coordinate frames, respectively.

For free vectors, we add superscript *N* or *S* to denote whether they are measured in navigation or sensor frames, and subscripts *x*, *y*, or *z* to denote their coordinates.

The orientations of the sensors are estimated by fusing measurements of a gyroscope ($y^{g}$), an accelerometer ($y^{a}$), a magnetometer ($y^{m}$) and reference values such as Earth’s gravity vector ($g^{N}$) and magnetic field vector ($m^{N}$). The main idea is to combine high-frequency angular rate information in a complementary manner with accelerometer and magnetometer data through the use of a sensor fusion algorithms such as complementary [10] or Kalman filters [11,12,13,14,15].

The gravitation vector is denoted by *g* and the magnetic field vector is denoted by *m*. In computations, the length of the gravitation vector is $∥g∥=9.81$ and the magnetic field vector length is normalised to one, $∥m∥=1$. Consistently to our notational convention, we add superscript *N* or *S* to indicate coordinate frames in which these vectors are expressed (measured). Consequently, $g^{N}$, $m^{N}$, $g^{S}$ and $m^{S}$ denote, respectively, gravitation and the magnetic field vectors measured in the navigation frame *N* and the sensor frame *S*. Vectors $g^{N}$, $m^{N}$ are constant and known. Their coordinates are

(1) $$g^{N}={\left[0\;0\;-9.81\right]}^{T}$$

and

(2) $$m^{N}={\left[\cos\left(\varphi^{L}\right)\;0\;-\sin\left(\varphi^{L}\right)\right]}^{T},$$

wherein $\varphi^{L}$ is the geographical latitude angle. The experiments were performed in a laboratory characterised by geographical latitude angle of $\varphi^{L}=66^{\circ }=1.1519$ rad.

The outputs of the successive sensors can be described by the following equations:

**Magnetometer:**

(3) $$y^{m}=m^{S}+error$$

**Inertial sensor (accelerometer):**

(4) $$y^{a}=a^{S}-g^{S}+error$$

**Gyroscopic sensor:**

(5) $$y^{g}=\omega^{S}+error$$

If necessary, the dependence of measurements on time is additionally marked, by $y^{m}\left(t\right)$, $y^{a}\left(t\right)$, $y^{g}\left(t\right)$ (continuous time) or by adding the subscript *k*, $y_{k}^{m}$, $y_{k}^{a}$, $y_{k}^{g}$ (discrete time).

The relative orientation between navigation and sensor coordinate frames is defined by a 3×3 rotation matrix *R*. Consequently, navigation and sensor coordinates of a given free vector *v* are related by

(6) $$v^{N}=Rv^{S}.$$

Rotation matrix *R* can be represented/parameterized in several ways. The most common is parametrization by the usage of Euler angles and parametrizations using unit quaternions ($q={\left[q_{0},q_{1},q_{2},q_{2}\right]}^{T}$, where ${\left[q_{1},q_{2},q_{3}\right]}^{T}\epsilon R^{3}$ is vector part). Parametrizations make for easier operations on orientations and are more efficient numerically.

## 4. Sensors Evaluation

There are several different varieties of IMUs. One of the more common types is defined by being manufactured using microelectromechanical system (MEMS) technology. The MEMS IMUs cost from several to several hundred euros depending on the performance, which makes them a popular choice among electronics developers. They are commonly used in modern mobile phones, unmanned aerial vehicles (UAV) and the automotive industry. However, due to the imminent noise present in any type of sensor, IMUs can never produce perfect measurements of acceleration, angular velocity and intensity of the magnetic field.

The commercial grade MEMS accelerometers have a significant bias error (typically, more than 60 mg). On the other hand, commercial MEMS gyroscopes measurements are affected by considerable Angular Random Walk (often greater than 50 (deg/$\sqrt{hr}$)). The MEMS magnetometers are sensitive to magnetic interference. Moreover, the respective calibration process is needed to obtain reliable measurements. The final result is that the estimates of linear velocity, position and orientation based directly on those measurements without any filtration become inaccurate and unusable after a short period.

The quality of sensors plays the pivotal role in the results achieved by the system. Depending on their parameters, the devices can be qualified into different applicability areas such as commercial, tactical, navigation and strategic [28]. A similar classification is also applied in the industry: navigation (top performance), tactical, industrial, automotive (lowest performance) [29]. The detailed classification of the IMU according to performance is presented in Table 1.

In our tests we used three IMU devices - two from the external vendors: Fairchild Semiconductor FMT1030 [30] and Xsens MTi-1 [31], and an experimental, custom built one [26].

### 4.1. Materials and Methods

The purpose of this experimental stage was to evaluate the candidate sensor devices and select the most appropriate ones for the designed application. The first problem in the evaluation of the IMU class devices is to define what shall be measured and what are the quality criteria. There are two possible approaches which can be found in the literature—a measurement of calculated orientations or raw sensor values. The first approach is usually employed to evaluate the outcome of a system as a whole (i.e., motion capture or navigation), although sometimes it is employed for the evaluation of sensor devices as well (e.g., [32,33]). The second approach dominates in the analysis of low-level hardware.

Using computed orientations at this stage seems unwise since their calculation involves entirety of processing pipeline and the results might be affected at any stage of the processing pipeline, by an influence that is hard to identify since all other processing stages contribute to the overall quality.

The other approach, evaluating raw sensor values, requires comparing them to the reference ones. Ground truth values can be obtained from the optical motion capture system. It is relatively easy to obtain the ground truth for the gyroscope as angular velocity is just a first derivative of the orientation. However, obtaining the acceleration in the sensor coordinate system requires obtaining the orientation of the device, which can be drift-affected. The other problem is obtaining true magnetic field induction in magnetometers which would require both orientation of the device and reference 3-axial magnetometer.

In order to avoid establishing ground truth values, which is troublesome, the authors proposed performing a static test with known zero motion as a ground truth. Furthermore, Allan variance (AVAR) was employed for this test, which has a well defined interpretation for certain parts of its plot and allows for both qualitative and quantitative description. It is defined as follows:

(7) $$\sigma_{y}^{2}\left(\tau \right)=\langle \frac{1}{2}{\left(y\left(t+\tau \right)-y\left(t\right)\right)}^{2}\rangle ,$$

wherein brackets 〈〉 denote expected value.

**Figure 3:**
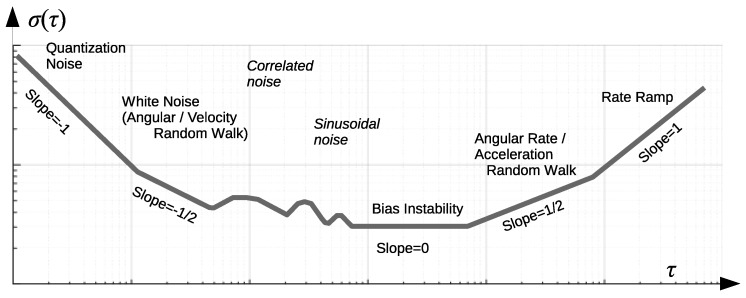
Schematic overview of Allan deviation plot interpretation.

Allan variance and its square root, Allan deviation, introduced in [34] is a standard method of characterization of noise in IMU devices [35,36,37]. While other methods, namely the power spectral density distribution and autocorrelation function, can provide similar information, their interpretation is not as clear and intuitive as it is with Allan variance. Seven distinct types of noise (see Figure 3) which contribute to the overall drift, can be recognized by observing the log-log plot of Allan deviation vs. cluster time *τ* of which five are easily obtainable with a plot readout [28,38]. A subset of these parameters is usually provided in the device characteristics by the device vendors.

A schematic plot is demonstrated in Figure 3. The horizontal axis of the AVAR plot is oriented in reverse to the frequency. The high-frequency noise is located to the left, whereas the low frequencies are located to the right, these are respectively short and long *τ* intervals. The quantization noise (QN) is visible leftmost in the plot, characterised by a slope of −1. A line fitted to the actual data evaluated at the $\tau =\sqrt{3}$ cluster time defines the quantization coefficient. This component is not always observable in the actual data. Next, the white noise term contribution can be seen in the plot due to integration to gyroscope’s angle or accelerometer’s velocity random walk (ARW/VRW). The slope being $-\frac{1}{2}$ is strictly related to this type of noise. A fitted line evaluated at τ=1 cluster time defines the random walk coefficient. Once the curve reaches the minimum, a bias instability can be read, which is the result of a low-frequency random flickering in the electronics used. A line with the slope of 0 is fitted to the points defining local minima, which after appropriate scaling by 2ln2 gives the value of bias instability (BI). Similarly to the random walk, the rate random walk (RRW) can be read after fitting a line of slope $+\frac{1}{2}$ and finding its intersection with τ=3. The slope of +1 read from the regression line at $\tau =\sqrt{2}$ indicates the rate ramp (RR). Two additional noise types are hardly characterised using AVAR. One can find an exponentially correlated noise if both slopes of $+\frac{1}{2}$ and then $-\frac{1}{2}$ are present one after another, although its presence is not as clear in practice, similarly to sinusoidal noise (repeated +1/−1 slopes), which might be occluded.

### 4.2. Experimental Results

The Allan variances for all the considered sensors were obtained experimentally with a static recording. All the tested sensors were attached to the same rigid body, which was placed on the sheets of foam with different hardness structures. The foam acts as a damping filter to eliminate the vibrations of the surrounding environment. The experiment was conducted in the rural area on the ground level on the concrete floor. There were no significant metal objects in the vicinity of the measurement point so that none could affect the operation of the magnetometer. The recordings were performed simultaneously, with 100 Hz sampling and took over 5 h.

**Figure 4:**
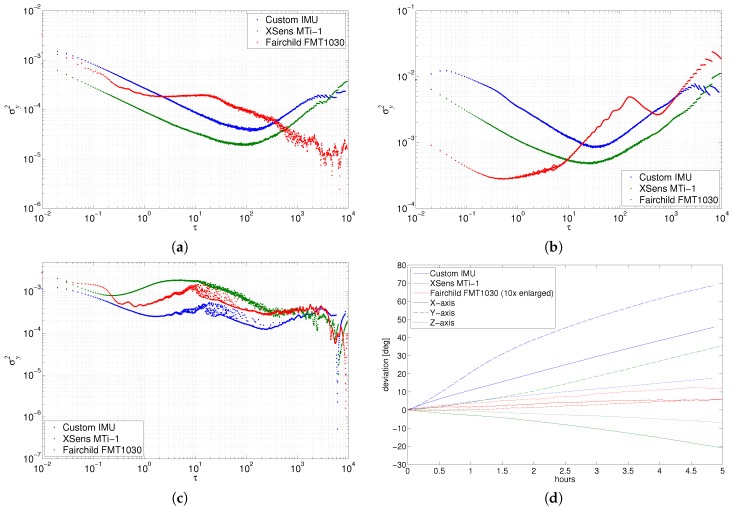
Static experiment results: representative Allan variances for the tested IMUs: (**a**) gyroscopes; (**b**) accelerometers; (**c**) magnetometer (for yaw axis-Z); (**d**) drift resulting from gyroscope integration.

The representative results are shown in Figure 4a–c. It appeared that the commercial Xsens IMU outperformed our custom one, as it returned slightly lowered AVAR curves for the gyroscope and the accelerometer, although they attained similar characteristics. While these two devices returned typical results, the FMT1030 provided non-typical outcomes.

For the gyroscope, no QN or RR were observed for any device. The white frequency noise term contributing to ARW and VRW can be seen in the beginning plot of Figure 4a for all the devices. The rate random walk was also observed for custom IMU and Xsens, but it wasn’t present in the FMT1030 characteristics. The most notable difference regards BI: it is clearly observed in Xsens and Custom IMU, but it is ambiguous for the gyroscope of the FMT1030 device, where it is uncertain whether the curve is flattening roughly in the middle of the plot or it is located nearer the right side of the plot. The latter would be beneficial as it means the appearance of the BI for the long times (hours).

Similar results are also visible for the accelerometer. Xsens and custom devices behave like typical devices with VRW, BI and RRW clearly identifiable. The most notable results are in the nontypical characteristics of the FMT device. For the short observation times, on the left side of the plot Figure 4b, from single samples to seconds, it has very low $\sigma_{y}^{2}$ values—an order of magnitude lower than the other two. For the long observation times of FMT1030, one can observe untypical RRW with bump and valley in the plot—probably some periodic distortion.

The magnetometer outcomes of all devices are on par with each other (Figure 4c), with unclear identification whether the noise is white noise or rather bias instability. Generally, Xsens magnetometer returned slightly higher $\sigma_{y}^{2}$ values than FMT’s and even higher than custom IMU’s magnetometer.

### 4.3. Conclusions

The most unexpected and ambiguous results are observed for FMT1030 which differs significantly from the other two. The effect of internal processing is clearly visible in the integration results (Figure 4d), which indicates that similar results can be achieved for other sensors if the processing of the signal is designed with the noise characteristic in mind.

The other conclusion is that it seems the modern IMU with its complex internal signal processing, designed to suppress different kinds of noise, can be hardly characterised by a simple coefficient reading from the plot as it is described in the previous paragraph. On the other hand, AVAR is still a useful tool for a visual examination. One can compare the noise characteristics of devices for certain time ranges when due to a malformed shape of AVAR curve, automated interpretation or when the parameter estimation is not possible. A good example of such a visual interpretation is demonstrated in Figure 4a,b, where the FMT1030 gyroscope has a notably lower random walk noise for the longer time ranges (above 200–300 s) than the other devices, and the FMT1030 accelerometer notably outperforms competing devices for the short time ranges (below one second). Such a characteristic is very desirable as the error is lowered for each of the sensors within the applicability range—as it is explicitly used in a complementary filter.

## 5. Sensor’s Orientation Estimation

### 5.1. Materials and Methods

The three implemented and evaluated algorithms for the estimation of the relative orientation of the moving body based on measurements of triaxial angular rate sensors, accelerometers, and magnetometers use basic relations for the kinematics of the orientation change. Here, we consider only the orientation estimation in a free segments model, without a kinematic chain model [19,39,40]. In this section, we present constructions of these three algorithms with the use of the introduced notations.

Due to the motion of the body, the rotation matrix *R*, the unit quaternion *q* and Euler angles yaw $\phi_{y}$, pitch $\phi_{p}$ and roll $\phi_{r}$ are functions of time, i.e., R=R(t), q=q(t) and $\phi_{ypr}=\phi_{ypr}\left(t\right)$. Mathematical models for the evolution of rigid body orientation with time can be formulated for all parameterizations of the rotation matrix in the form of differential equations. For rotation matrix R(t), the differential equation for motion has the form presented in Equation (8).

(8) $$\frac{d}{dt}R\left(t\right)={\left[\omega^{N}\right]}_{\times }R\left(t\right)=R\left(t\right){\left[\omega^{S}\right]}_{\times }$$

wherein ${\left[\omega^{N}\right]}_{\times }$ and ${\left[\omega^{S}\right]}_{\times }$ are 3×3 cross - product matrices.

For parameterization of orientations defined by unit quaternions q(t), the differential equation for motion is presented in Equation (9).

(9) $$\frac{d}{dt}q\left(t\right)=\frac{1}{2}\omega^{N}⊗q\left(t\right)=\frac{1}{2}q\left(t\right)⊗\omega^{S}$$

When using orientation representation by yaw, pitch and roll angles $\phi_{y}\left(t\right)$, $\phi_{p}\left(t\right)$, $\phi_{r}\left(t\right)$, the differential equation for their time evolution is presented in Equation (10).

(10) $$\frac{d}{dt}\phi_{ypr}\left(t\right)=M\left(\phi_{ypr}\left(t\right)\right)\omega^{S}$$

wherein $M(\phi_{ypr})$ is a matrix with entries dependent on angles $\phi_{ypr}$.

In practical implementations, estimators of orientations are realised on the basis of digital systems. Therefore, in descriptions of algorithms in the forthcoming subsections equations for kinematics of orientations changes are replaced by their discrete counterparts. Discrete time index is denoted by the subscript *k*.

All constructions of the orientation estimators can be interpreted as complementary filters in the sense that they perform a fusion operation of output signals of sensors. We prefer to work on quaternions to avoid problems with rotation order and Gimbal lock singularities. We have chosen three different algorithms based on the quaternion representation of orientation. Such algorithms are representative of the three groups of different ways to handle the external acceleration because the existence and magnitude of the external acceleration of the IMU sensor strongly influence the orientation estimation results. We also introduced unified notation for all parametrizations of the estimated filter orientations, namely by a hat symbol ($\hat{q},\hat{R}$).

#### 5.1.1. Nonlinear Complementary Filter on SO(3) (NCF)

The first implemented algorithm for orientation estimation is one of the versions of filters described in [10] (SO(3) denotes a special orthogonal group of rotations). The idea behind the construction of the orientation estimator is the modification of the equation of kinematics of motion (Equation (8)) by using the nonlinear feedback based on the instantaneous estimate of the relative rotation.

An algorithm of “instantaneous” estimates the relative rotation $\overset{ˇ}{R}$ between the coordinate frames *N* and *S* using two nonparallel vectors *g* and *m* and their measurements in two coordinate frames, navigation $g^{N}$, $m^{N}$, and sensor $g^{S}$, $m^{S}$. For quaternion representation of rotation $\overset{ˇ}{q}$, the estimate, based on the accelerometers and the magnetometers vector measurements $y^{a}$ and $y^{m}$ can be obtained by using the solution to the Wahba problem [41,42,43].

The equation for dynamics of the orientation estimate, with nonlinear feedback has the form presented in Equation (11) (as in Equation (10) in [10]).

(11) $$\frac{d}{dt}\hat{R}\left(t\right)=\hat{R}\left(t\right){\left[y^{g}+k_{p}\tilde{\omega }\right]}_{\times }$$

wherein ${\left[.\right]}_{\times }$ again denotes the cross product matrix, $k_{p}$ is the feedback gain and $\tilde{\omega }$ is the nonlinear term corresponding to the error between the filter estimate $\hat{R}$ and the instantaneous estimate of the rotation matrix $\overset{ˇ}{R}=\overset{ˇ}{R}\left(y^{a},y^{m}\right)$.

Equation (11) can be equivalently formulated with the use of the quaternion representation of the orientation, as in Equation (12).

(12) $$\frac{d}{dt}\hat{q}\left(t\right)=\frac{1}{2}\hat{q}\left(t\right)⊗\left(y^{g}+k_{p}\tilde{\omega }\right)$$

The quaternion formulation is more convenient for computational implementation. The implementation of the nonlinear, complementary filter involves a discretized version of Equation (12), which is presented as Equation (13).

(13) $${\hat{q}}_{k+1}=\left[I+\frac{\Delta t}{2}M_{R}\left(y^{g}+k_{p}\tilde{\omega }\right)\right]{\hat{q}}_{k}$$

wherein *I* is the 4×4 identity matrix, $M_{R}\left(y^{g}+k_{p}\tilde{\omega }\right)$ stands for the matrix representation of the quaternion right multiplication by the pure quaternion $y^{g}+k_{p}\tilde{\omega }$ and Δt is the sampling interval.

**Figure 5:**
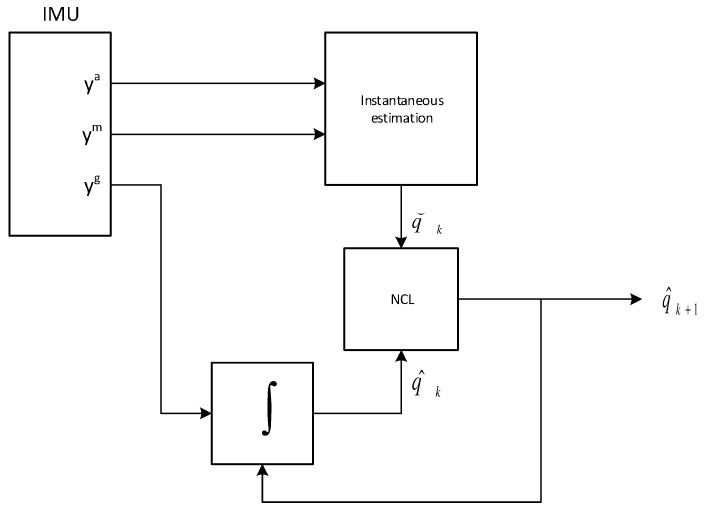
The NCF flow chart.

The matrix exponential function could also be used in Equation (13). For small values of sampling interval Δt they are practically equivalent. Iterations are additionally augmented by normalizing the estimated quaternion in each iteration. The initial condition is a unit quaternion ${\hat{q}}_{0}$, converted from the instantaneous estimated rotation matrix $\overset{ˇ}{R}\left(y_{0}^{a},y_{0}^{m}\right)$. The pseudo code using the Matlab quaternion functions is presented as Algorithm 1 and a flow chart is in Figure 5.

 **Algorithm 1:** Nonlinear complementary filter pseudo code (Matlab style)**Data**: $q_{k-1}$, $y_{k}^{a}$, $y_{k}^{m}$, $y_{k}^{g}$**Result**: $q_{k}$**Parameter**: $g^{N}$, $m^{N}$, $k_{p}$, Δt$q_{k}=\left[0,y_{k}^{g}\right];$$q_{inst}=calcInstantaneous\left(y_{k}^{m},y_{k}^{a},m^{N},g^{N}\right);$$q_{residual}=quatmultiply\left(quatconj\left(q_{k-1}\right),q_{inst}\right);$$om_{residual}=0.5*\left(q_{residual}-quatconj\left(q_{residual}\right)\right);$$q_{k}=0.5*\Delta t*uatmultiply\left(q_{k-1},\left(q_{k}+k_{p}*om_{residual}\right)\right)+q_{k-1};$$q_{k}=quatnormalize\left(q_{k}\right);$return $q_{k}$;


#### 5.1.2. Extended Quaternion Kalman Filter (EQKF)

The second algorithm for the orientation estimation, presented in [11], is based on the idea of the construction of the extended quaternion Kalman filter for the analysed process model. An orientation of the sensor is represented by using the quaternion parameterization. The designed filter adopts a two-layer filter architecture in which the first block uses an instantaneous estimate of the orientation. The second block is the extended Kalman filter. The Kalman filters consist of the following blocks: process model, measurements model, time update (prediction based on process model) and measurement update (correction based on measurements) [44].

The construction of the extended Kalman filter is based on the model of orientation kinematics with seven-dimensional state vector *x*, where the first three components are defined by the angular rate vector $\omega^{S}$, and the last four components are the sensor orientation quaternion *q*.

(14) $$x=\left[\begin{array}{c} \omega^{S}\\ q \end{array}\right]$$

With the state vector (Equation (14)) the differential equation for orientation kinematics is as shown in Equation (15).

(15) $$\frac{d}{dt}x=f\prime \left(x\right)=\left[\begin{array}{c} \frac{1}{\tau }\left(\omega^{S}+w\right)\\ \frac{1}{2}q⊗\omega^{S} \end{array}\right]$$

The above formula is a filter process model (see Figure 1 in [11]). The first block defines time evolution of the angular velocity $\omega^{S}$, modelled as a coloured noise generated by a linear inertial system with a white noise input *w* and the time constant *τ*. The second block, involving orientation quaternion *q*, is equivalent to Equation (9). It should be noted that Equation (15) is a system of non-linear differential equations.

Equation (16) is the measurement equation based on a state vector.

(16) $$\hat{z}=\hat{x}+v,$$

And the measurement is as follows:

(17) $$z_{k}=\left[\begin{array}{c} y_{k}^{g}\\ {\overset{ˇ}{q}}_{k}\left(y_{k}^{a},y_{k}^{m}\right) \end{array}\right]$$

The lower block in the measurement *z* is an instantaneous estimate of the orientation, represented by a unit quaternion, denoted by $\overset{ˇ}{q}\left(y^{a},y^{m}\right)$. In our implementation, the Quest method [42] is used.

The extended Kalman filter algorithm is based on the discretization and the linearization of a nonlinear process model (Equation (15)) relating to the estimated trajectory of the process.

**Figure 6:**
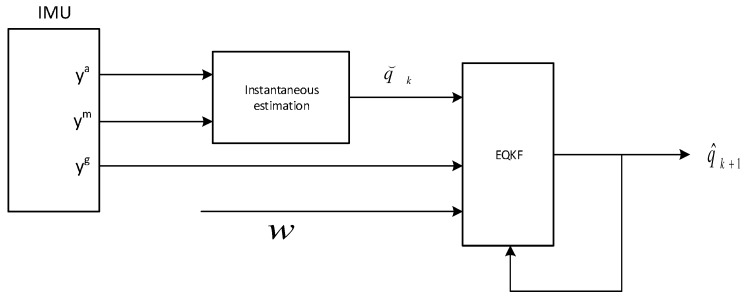
The EQKF flow chart.

The covariance matrix *V* of the measurement error *v* is assumed in Equation (18)

(18) $$V=\left[\begin{array}{cc} \sigma_{g}^{2}\cdot I_{3\times 3} & 0_{3\times 4}\\ 0_{4\times 3} & \sigma_{q}^{2}\cdot I_{4\times 4} \end{array}\right]$$

where diagonal elements $\sigma_{g}^{2}$ are a variance of the angular rate measurements and $\sigma_{q}^{2}$ is a variance experimentally determined based on a computed quaternion by the Quest method. Here we describe only the most important elements of the filter implementation, the whole Kalman filter equations can be found in [11]. The flow chart of the filter is presented in Figure 6. The pseudo code using the Matlab quaternion functions is presented as Algorithm 2.

 **Algorithm 2:** Extended quaternion Kalman filter pseudo code (Matlab style)**Data**: $x_{k-1}$, $y_{k}^{a}$, $y_{k}^{m}$, $y_{k}^{g}$, $P_{k-1}$**Result**: $q_{k}$**Parameter**: $g^{N}$, $m^{N}$, Δt, *τ*, *Q*, *V*% *x* is a 7×1 state vector% *Q* is a set covariance matrix of process noise% *V* is a set covariance matrix of measurement noise% *P* is a state error covariance matrix% *f* a process model function% *H* is a set 7×7 identity matrix (measurement model is linear)% Φ is a state transition matrix, this is the Jacobian matrix of partial derivatives of the state function f% projection equations$\Phi_{k}=clacPhiMatrix\left(x_{k-1},\Delta t,\tau \right)$;$x_{k}=f\left(x_{k-1},\Delta t,\tau \right);$$P_{k}=\Phi P_{k-1}\Phi^{T}+Q$;% Kalman gain computation$K=P_{k}H^{T}{\left(HP_{k}H^{T}+V\right)}^{-1}$;% create model measurement vector$\hat{z}=Hx_{k}$;% create sensor measurement vector$q_{inst}=calcInstantaneous\left(y_{k}^{m},y_{k}^{a},m^{N},g^{N}\right);$$z=[y_{k}^{g};q_{inst}]$;% update equations$x_{k}=x_{k}+K\left(z-\hat{z}\right)$;$P_{k}=\left(I-KH\right)P_{k}$;$q_{k}=quatnormalize\left(x{\left(4:7\right)}_{k}\right);$return $q_{k}$;


#### 5.1.3. Adaptive Extended Quaternion Kalman (AEQKF)

The last algorithm implemented is an adaptive extended quaternion Kalman filter (AEQKF), presented in [13], without augmented biases in a state vector:

(19) $$x=\left[\begin{array}{c} q \end{array}\right]$$

The process model is based on a discrete-time version of Equation (9) with angular velocity values $\omega_{k}^{S}=y_{k}^{g}$ as control inputs.

The measurements are the output from the accelerometer and the magnetometer, as in Equation (20). The measurement model $\hat{z}$ estimates the acceleration and the magnetic vector by the rotation references vectors Equation (21). Here we describe only the most important elements of the filter implementation, the whole Kalman filter equations can be found in [13].

(20) $$z_{k}=\left[\begin{array}{c} y_{k}^{a}\\ y_{k}^{m} \end{array}\right]$$

(21) $${\hat{z}}_{k}=h\left({\hat{x}}_{k}\right)+v=\left[\begin{array}{cc} R(q_{k}) & 0\\ 0 & R(q_{k}) \end{array}\right]\left[\begin{array}{c} g^{N}\\ m^{N} \end{array}\right]+\left[\begin{array}{c} v_{k}^{a}\\ v_{k}^{m} \end{array}\right]$$

Wherein R(q) is a rotation matrix defined by the quaternion *q*.

As described in [13], the model of the measurement process is used for a construction of the adaptation mechanism of the estimator. Namely, it is assumed that the covariance matrix of the measurement error is changing in time; its values (magnitudes) are dependent on the estimate of the reliability of the measurements. There are two adaptation mechanisms assumed in [13], one for accelerometers and another for magnetometers. Here we only implement adaptation regarding the accelerometers measurement, where a covariance matrix of the measurement $V_{k}$ depends on the deviation of the value of the gravitational acceleration $∥g∥$ and the measured acceleration magnitude $∥y_{k}^{a}∥$, as in Equations (22) and (23).

(22) $$V_{k}=\left[\begin{array}{cc} \sigma_{a}^{2}\cdot I_{3\times 3} & 0_{3\times 3}\\ 0_{3\times 3} & \sigma_{m}^{2}\cdot I_{3\times 3} \end{array}\right]$$

if $\left|∥y_{k}^{a}∥-∥g∥\right|<\epsilon$ or

(23) $$V_{k}=\left[\begin{array}{cc} \infty \cdot I_{3\times 3} & 0_{3\times 3}\\ 0_{3\times 3} & \sigma_{m}^{2}\cdot I_{3\times 3} \end{array}\right]$$

otherwise. Wherein $\sigma_{a}^{2}$ is a variance of the accelerometer and $\sigma_{m}^{2}$ are magnetometer measurements.

**Figure 7:**
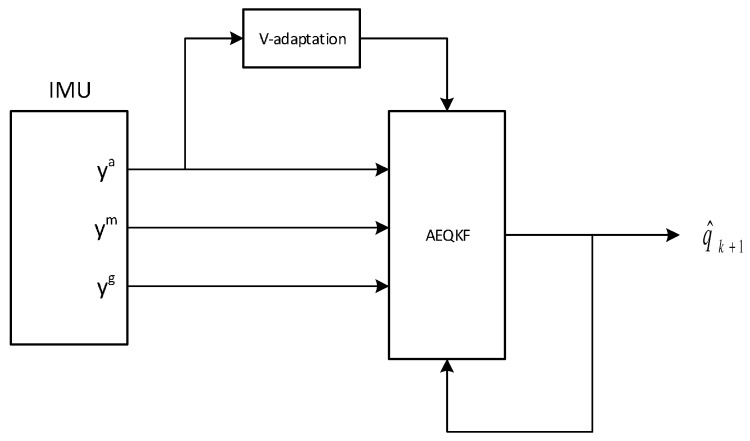
The AEQKF flow chart.

The pseudo code using the Matlab quaternion functions is presented as Algorithm 3. The flow chart is shown in Figure 7.

 **Algorithm 3:** Adaptive extended quaternion Kalman filter pseudo code (Matlab style)**Data**: $x_{k-1}$, $y_{k}^{a}$, $y_{k}^{m}$, $y_{k}^{g}$, $P_{k-1}$**Result**: $q_{k}$**Parameter**: $g^{N}$, $m^{N}$, Δt% *x* is a 4×1 state vector% *Q* is a covariance matrix of process noise% *V* is a covariance matrix of measurement noise% *P* is a state error covariance matrix% *f* a process model function% *h* a measurement model function% *H* is a measurement model matrix, this is the Jacobian matrix of partial derivatives of the measurement function h% Φ is a state transition matrix, this is the Jacobian matrix of partial derivatives of the state function f% projection equations$\Phi_{k}=clacPhiMatrix\left(x_{k-1},y_{k}^{g},\Delta t\right)$;$x_{k}=f\left(x_{k-1},y_{k}^{g},\Delta t\right);$$Q=calcQMatrix(y_{k}^{g},\Delta t);$$P_{k}=\Phi P_{k-1}\Phi^{T}+Q$;% V matrix adaptation$V=calcVMatrix(g^{N},y_{k}^{a});$% Kalman gain computation$K=P_{k}H^{T}{\left(HP_{k}H^{T}+V\right)}^{-1}$;% create model measurement vector$H=calcHMatrix(x_{k},g^{N},m^{N})$;$\hat{z}=h\left(x_{k},g^{N},m^{N}\right)$;% create sensor measurement vector
$z=[y_{k}^{a};y_{k}^{m}]$;% update equations$x_{k}=x_{k}+K\left(z-\hat{z}\right)$;$P_{k}=\left(I-KH\right)P_{k}$;$q_{k}=quatnormalize\left(x_{k}\right);$return $q_{k}$; 


#### 5.1.4. Experiment

To verify the performance of the IMU-based system experimentally, we propose the use of the representative series of motion sequences recorded using IMU sensors, along with the reference that one acquired with the optical motion capture system. The choice of an optical system was based on the fact that orientations obtained using the optical motion capture offer very fine accuracy and precision and are drift proof due to the lack of the iterative integration of velocity or acceleration.

**Figure 8:**
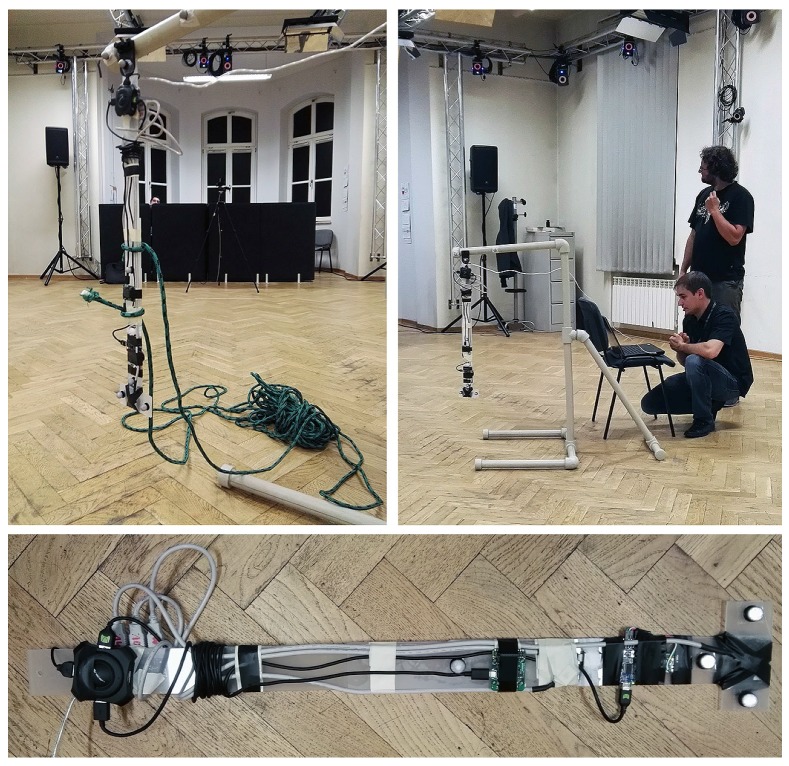
The pendulum with attached IMUs during the tests in the HML - motion capture laboratory.

The recordings were performed at the Human Motion Lab (HML) of PJATK (Poland). The acquisition employed Vicon Nexus 2.3 working with 3×10 Vicon NIR cameras (MX-T40, Bonita 10 and Vantage 5). All the recordings were performed at 100 fps. A single segment pendulum of 70 cm length (see Figure 8) was used as a model. It was swinging in one axis, although the suspension was flexible, so the side wobbling or motions were also available. According to the Vicon reference [45], the certified accuracy of the marker location using Vantage camera set is 1mm, standard deviation measuring the precision is below 1mm. Assuming the two markers, necessary for the identification of the attitude, are located at the ends of the considered pendulum, the reference system’s results are in maximal angular inaccuracy of 0.16° and angular imprecision expressed with a standard deviation is below 0.11°. Furthermore, actual precision and accuracy of the reference system are better than declarative.

For testing purposes, we prepared a comprehensive set of recordings simulating movement similar to gait and manual operations, the details are presented in Table 2. The experimental data are also available in our repository, the RepoIMU (http://zgwisk.aei.polsl.pl/index.php/en/research/projects/61-repoimu) [46].

#### 5.1.5. Synchronization Problem

Another issue to be addressed when compiling data is synchronisation. Since clocks in various devices are of a different quality, the synchronisation is a necessary step to be performed. The key issue is that we have neither a common clock nor a common coordinate system for the measures, so there is no easy base to align the sequences. The problem was addressed in [33] using the principal component analysis (PCA) matching using the dynamic time warping (DTW) synchronisation. Alas, as one can consider DTW as a denoising filter, we proposed our own orientation synchronisation method PCA-TSA (PCA based Two Step Alignment) [47], which is based on the block matching of the (quaternion) PCA signals. The method was later adopted [46] to match raw IMU measures using the linear PCA of the angular velocities from the gyroscopes. The key points outlining the synchronisation are as follows:

1. First, the PCA on the angular velocities is employed to get as much motion as possible in a single dimension. Thanks to this, all further steps are completely agnostic to the coordinates rotation.
2. Then, we match the beginnings and the ends using block matching, and we trim the protruding parts outside of the common time range.
3. Next, the secondary alignment, span a pool of nodal points in a reference sequence and match the corresponding points using the surrounding block matching with a sum of absolute difference.
4. Using reference time in known nodal points, we interpolate time for the matched sequence.
5. The matched time series is interpolated with the new time using 1D linear interpolation or using a phase of complex numbers for the orientation angles.
6. The rotation between the coordinate systems is identified as an average angular difference between the two sequences.

When using the synchronisation method, one must decide what the reference timer is and what is to be matched. The synchronisation of the reference orientations to the timer of the IMU controlling computer, as in the [46], makes sense in our case as we want to get true raw sensor values with corresponding true orientations in the respective moments. The opponent approach, to synchronise the resulting final orientations obtained with fusion filters to the reference system, would make sense if we would like to evaluate the system as a whole, but excluding temporal discrepancies.

#### 5.1.6. Estimation Measures

The evaluation of performances for the orientation estimation algorithms is done on the basis of average deviations between true and estimated orientations of the sensor. Deviation index (measure) between true and estimated orientations can be, however, defined in different ways [48].

For orientation results in quaternions, we can use the deviation index $DI_{Q}$ corresponding to the geodesic distance between two quaternions - filter estimate $\hat{q}$ and the true rotation *q* from the Vicon system, on the hypersphere $S^{3}$:

(24) $$DI_{Q}=2*arccos(|\hat{q}*q\left|\right)$$

All evaluations and comparisons of performances of algorithms for the orientation estimation are based on quaternion deviation indexes $DI_{Q}$ averaged over the experiment time horizon.

### 5.2. Results

Filter parameters are following:

- parameter Δt=0.01,
- parameter $k_{p}$ in complementary filter (NCF): $k_{p}=2$,
- parameters of EQKF filter: τ=0.5, $\sigma_{g}^{2}=0.001$, $\sigma_{q}^{2}=0.00001$ used in the measurement matrix,
- parameters of AEQKF filter for the adaptation measurement covraiance matrix: ϵ=0.4, $\sigma_{a}^{2}=0.001$ and $\sigma_{m}^{2}=0.00001$.

**Figure 9:**
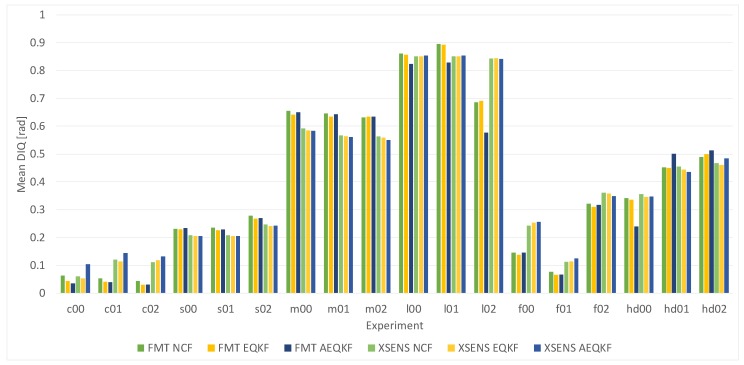
The mean deviation index $DI_{Q}$ for experiments (details in Table 2).

The average $DI_{Q}$ index for the three implemented orientation filters NCF, EQKF and AEQKF in every scenario is presented in Figure 9. The results are computed based on signals from FMT and Xsens sensors. The Custom IMU was not included in the tests on the pendulum, because of the worst results obtained in the previous test.

### 5.3. Discussion

It is well known that a factor that strongly influences the orientation measurement is the existence and magnitude of the external acceleration of the IMU sensor. For more dynamic motion we obtained larger error values (scenario *m00-m02*, *l00-l02*, and *hd00-hd02*). This is consistent with our previous results presented in [49].

In the NCF filter, it is assumed that external acceleration of the sensor is either not present, its magnitude is negligible compared to the gravity acceleration, or that the external acceleration is constant. Therefore, for this filter we get less favourable results. The best results are for the adaptive orientation estimation filter (AEQKF), where some auxiliary computations are performed in order to estimate the temporary magnitude of the external acceleration and then, the weight for the accelerometer channel is adaptively changed on the basis of the estimates.

There is no direct implementation of bias estimation in the mentioned filters, so for the sensor with better parameters (FMT sensor), we obtain smaller error for the orientation. The mean difference between orientations estimated on the basis of FMS and the Xsens measurements is about $4^{\circ }$ for AEQKF filter. The results provided by the FMT and Xsens sensors are on par, as it could be expected, as they share the hardware design and the key difference is in the built-in processing software.

A noteworthy observation was made for the recordings which were started with the pendulum hanging still for a short while before the main motion was started (scenario *c00-c01* and *f00-f02*). Apparently, the internal sensor filter was able to tune properly, so the results were significantly better than for the sequences which were started with immediate motion. This suggests that the two-fold Kalman filtering—hardware specific in the sensor package [30] and in software at the fusion stage—can improve the results if the former is properly initialized.

## 6. Conclusions

In this paper, a wearable inertial motion capture system has been presented. Its design is a complex problem due to the system’s many components. The selection of each component for every stage of the motion capture process significantly affects the final results of the system (final pose estimation). The authors have presented the selection procedure for the two key elements of the discussed system: state of the art sensors and algorithms for the sensor orientation estimation.

The results in Section 4 revealed that the Allan variance plot itself is useful as it provides a lot of information, though when facing modern sensors employing sophisticated filtering algorithms it requires a visual examination and interpretation. The final results of the system as a whole for the different sensors are coherent with the individual sensor test.

The FMT sensor, which had the most promising AVAR results, provided notably better results (on average) when the applied fusion algorithms could use an initial calibration (*c00-02* and *f00-02*) in a steady state. An average error angle is 30%–70% smaller than for the other sensor. It is consistent with the steady recording for the AVAR calculations which obviously allowed the sensor to calibrate. When the initial quiescence was not present, the Xsens sensor usually provided slightly better results, although that loss is relatively small, up to 15%.

## Figures and Tables

**Table 1:** Classification of the inertial sensors due to the application areas [28].

Application Grade	Gyroscope Performance	Accelerometer Performance
Commercial/Consumer	>1 deg/s	>50 mg
Tactical	∼1 deg/h	∼1
Navigation	0.01 deg/h	25 μg
Strategic	∼0.001 deg/h	∼1 μg

**Table 2:** Recorded scenarios of pendulum motion.

Type	Symbol	Description	Records	Approx. Duration
calibrated	c00..c02	start angle 0 then hand driven to 30° and released	3	60–100 s
	s00..s02	start angle 10°	3	115–120 s
swinging	m00..m02	start angle 30°	3	150–160 s
	l00..l02	start angle 45°	3	150–170 s
	f00	start angle 0, hand driven	1	70 s
free moves	f01	start angle 0, driven with soft stick	1	80 s
	f02	start angle 0, driven with 2 rope rig	1	100 s
dynamic	hd00..hd02	start angle 30°, manual bouncing in random moments	3	50 s

## Abbreviations

The following abbreviations are used in this manuscript:

IMU	Inertial Measurement Unit
MDE	Multimodal Data Environment
MEMS	Microelectromechanical System
AVAR	Allan variance
QN	Quantization Noise
(A/V)RW	(Angular/Velocity) Random Walk
BI	Bias Instability
RRW	Rate Random Walk
RR	Rate Ramp
NCF	Nonlinear Complementary Filter
EQKF	Extended Quaternion Kalman Filter
AEQKF	Adaptive Extended Quaternion Kalman
PCA-TSA	Principal Component Analysis based Two Step Alignment
HML	Human Motion Lab, PJATK, Bytom, Poland (http://bytom.pja.edu.pl/)
